# MicroRNAs from Liquid Biopsy Derived Extracellular Vesicles: Recent Advances in Detection and Characterization Methods

**DOI:** 10.3390/cancers12082009

**Published:** 2020-07-22

**Authors:** Rares Drula, Leonie Florence Ott, Ioana Berindan-Neagoe, Klaus Pantel, George A. Calin

**Affiliations:** 1 Department of Translational Molecular Pathology, The University of Texas MD Anderson Cancer Center, Houston, TX 77054, USA; gcalin@mdanderson.org; 2 Research Center for Functional Genomics, Biomedicine and Translational Medicine, Iuliu Hatieganu University of Medicine and Pharmacy, 23 Marinescu Street, 40015 Cluj-Napoca, Romania; ioana.neagoe@umfcluj.ro; 3 Department of Tumor Biology, Center of Experimental Medicine, University Medical Center Hamburg-Eppendorf, 20246 Hamburg, Germany; pantel@uke.de

**Keywords:** extracellular vesicle, liquid biopsy, miRNA biomarker, exosome

## Abstract

Liquid biopsies have become a convenient tool in cancer diagnostics, real-time disease monitoring, and evaluation of residual disease. Yet, the information still encrypted in the variety of tumor-derived molecules identified in biofluids has proven difficult to decipher due to the technological limitations imposed by their biological nature. Such is the case of extracellular vesicle (EV) encapsulated ncRNAs, which have gained traction in recent years as biomarkers. Due to their resilience towards degrading factors they may act as suitable disease indicators. This review addresses the less described issues in this context. We present an overview of less investigated biofluids that can be used for EV isolation in addition to different isolation approaches to overcome the technical challenges these specimens harbor. Furthermore, we summarize the latest technological advances providing improvement to ncRNA detection and analysis. Thereby, this review summarizes the current state-of-the-art methodologies regarding EV and EV derived miRNA analysis and how they compare to current approaches.

## 1. Introduction

Liquid biopsies have become standard procedures in the diagnosis and evaluation of many malignancies, making them powerful tools in diagnostics, evaluation of therapeutic efficiency, and minimal residual disease assessment [1,2]. The strong point of liquid biopsies is in their minimal invasiveness as compared to classical excisional biopsies, providing accessible means for real-time evaluation of the disease. Therefore, research in the field of liquid biopsies aims to maximize the available information regarding the disease by the use of specific biomarkers, and reliable non-invasive disease indicating biomolecules could standardize and facilitate screening procedures and therapeutic approaches in cancer [3].

Currently, blood is the main investigated biofluid for the evaluation of circulating tumor-derived material, ranging from circulating tumor cells (CTCs), extracellular vesicles such as exosomes, to various cell-free biomolecules in the form of circulating tumor DNA (ctDNA), cell-free RNA (cfRNA), and proteins [4]. Isolation, detection, and characterization of CTCs can provide crucial information regarding disease stratification at the point of diagnosis, based on the mutational status of the tumor, chromosomal abnormalities, methylation status, and overall, the heterogeneity and complexity of the tumor. Yet, the clinical use of CTCs has been hindered by both technical and biological limitations [5,6]. The main technical limitation is the partial efficiency of current CTC selective enrichment methods from blood. Biological limitations consist of the low amount of CTCs present in earlier stages of the disease [5]. An alternative is the detection of circulating cell-free DNA or circulating tumor DNA (ctDNA) that is released by tumor cells into the bloodstream either in the form of actively secreted cfDNA (free or encapsulated) [7] or as a result from apoptotic and/or necrotic tumor cells [8]. Detection methods are constantly being developed and improved to increase the sensibility and to lower the critical mass threshold from which ctDNA can be identified in blood. The main limitations in investigating ctDNA consist of the minimal amount that can be detected and contaminations from other non-tumor sources [9]. DNA released from apoptotic leukocytes is one of the main sources of contaminants, especially when taking into account the post-chemotherapeutic associated non-targeted cellular death [10]. Additionally, depending on the structure of the ctDNA, the half-time is very limited, being prone to degradation by the nucleases in the bloodstream [11].

## 2. miRNA as Biomarkers

MicroRNAs (miRNAs) have been extensively investigated as potential reliable biomarkers from liquid biopsies. The 19–25 bp long non-coding transcripts have been first been associated with oncogenic processes based on their modifications in expression rates observed in different cancer types [12]. Since then, a plethora of studies have attempted to standardize and characterize the non-coding transcriptome of different cancer types to offer suitable biomarker miRNA candidates [13]. Expression level alterations of miRNA can also indirectly reflect chromosomal abnormalities of their originating tumor cells, as a high number of microRNA gene loci are located within chromosomal fragile regions [14,15,16] which are prone to translocations [17,18], amplifications [19], or deletions [20]. This, coupled with a complete mapping of miRNA loci in the genome and the evaluation of their enrichment in different biological fluids, can prove useful tools in evaluating disease status when ctDNA or CTCs are not available. The biomarker potential of circulating miRNAs has been the focus of many research groups in recent years that provided consolidating evidence for their implications in oncogenic processes and of their use as biomarkers. Increasing numbers of circulating miRNAs have been identified as encapsulated in extracellular vehicles (EVs) [21], adding a layer of complexity in their regulating abilities and role in cell communication [22]. Technical advantages in the use of circulating miRNA consist of the high resilience to RNase degradation and environmentally-based factors, extending the detection time since sample collection [23].

## 3. Cell-to-Cell Communication by Extracellular Vesicles

Extracellular vesicles consist of a class of membrane-bound vesicles of varying origin that can be secreted by a cell in different physiological conditions. One of the main types of EVs in which miRNA have been identified are exosomes, 30 to 150 nm-sized double-membrane vesicles that have been extensively studied due to their roles in cell-to-cell communication [24,25,26]. Exosomes originate from the endosome and are secreted following their assembly into multivesicular bodies (MVB). Thus, they contain a series of endosome specific makers, such as tetraspanins (i.e., CD9, CD63, and CD81) [27] and membrane surface components of the endosomal sorting complexes, such as ALIX, TSG101, and HSC70. Most of the beforementioned proteins act as universal exosomal markers, regardless of cell origin [28].

The internalization of the exosomes has been proven to influence recipient cells mainly due to the composition of the exosomal cargo [29]. “Signals” are encoded in the components of the exosomal cargo, consisting of fragmented nuclear DNA [30,31,32], mitochondrial DNA [33], proteins, growth factors [34] miRNAs [24,35], and long non-coding RNAs [36,37], making exosomes very important players in cell-to-cell communication. The exact mechanisms of how exosomal cargo is selected and loaded are still under constant discussion [38]. Yet, it has been proven that exosomes reflect, to some extent, the molecular profile of the originating cell, thus contributing to the propagation of the oncogenic phenotype [35,39,40,41]. Encapsulated nucleic acids have gained recent attention in the area of biomarker discovery firstly due to their presumed resilience to nuclease degradation, in comparison to free circulating DNA and RNA candidates [42]. Additionally, as mentioned previously, encapsulated genetic material might be an indicator of abnormalities in the originating cell’s processes. For example, studies on both normal and tumor cells indicate that the exosomal DNA loaded in the exosomes is primary cytoplasmic DNA that accumulated as a result of chemically-induced DNA damage [32] or cancer-associated chromosomal instability [43]. Yet, RNA can be more informative [44] and stable [42] for further analyses. The identification of exosomal miRNA provided novel insights into the therapeutic potential of exosomes [29,45]. As is the case with other exosomal cargo components, the selective loading of miRNA into exosomes has not been completely uncovered. Current evidence indicates that selective sorting of miRNA into exosomes is dependent on the early interaction between miRNA associated RISC complex proteins and MVB markers [46]. Other older studies pointed toward neutral sphingomyelinase-2 (nSMase-2) and ceramide production as a promoter of exosomal miRNA packaging [47]. The same group reported further evidence for nSMase-2 regulation of exosomal miRNA secretion, making the inhibition of nSMase2 a method of decreasing exosomal miRNA content [48].

## 4. The Potential Use of Exosomal miRNAs as Biomarkers

There is a current debate on whether EV/exosomal miRNAs (exomiRs) can be a more accurate indicator of disease in comparison to free circulating miRNA [49]. Most studies focused on exomiRs imply that the encapsulated miRNAs are less prone to degradation [42,50,51], making them more suitable biomarker candidates. Comparative evaluations of free circulating miRNA profiles and EV-derived miRNA profiles have not yet reached a consensus regarding their consistency, especially due to the lack of studies in which a comparison is included. Specifically, several studies point towards a great variation between the two: in prostate cancer, only a small fraction of the plasma identified miRNA was reflected in the exomiRs signature [52]. Yet, the expression levels of some exomiRs, such as let-7a-5p, was proven as more accurate criteria of differentiating prostate cancer patients based on the Gleason score. Similar results were reported by a group investigating the miRNA signature in the urine of healthy individuals. Out of the 184 identified miRNAs in the urine-derived exosomes, only seven miRNAs were identified in the cell-free urine, indicating either a selective packaging of the miRNAs or a higher degree of RNA degradation in urine [50]. One comparison showed that in EVs only a fraction of the miRNAs found in plasma could be detected. Reasons for that might be a general low abundance of miRNAs in EVs in this study and low efficiency of the EV isolation step. Nonetheless, they found different miRNAs as having better diagnostic potential either from plasma as well as from EVs which makes both valuable sources of miRNA markers [52]. This is further confirmed by a study [53] reporting the highest diagnostic accuracy in NSCLC when combining four serum miRNAs and two exomiRs [54]. In contrast, a study analyzed miRNA concentration in plasma, serum, and exosomes of three healthy volunteers and found on average a lower concentration of total miRNAs in plasma and serum compared to EVs after RNase A digestion, emphasizing the protective effect of the EV membrane in the bloodstream environment [55]. The limitation of this study is that it does not focus on miRNAs relevant to cancer which is why it does not necessarily mirror the situation in cancer patients, in which many studies have proven specific miRNA enrichment when compared to healthy individuals [56]. Yet, the observed differences between plasma and serum miRNA derived profiles and samples that have been processed by different methods are still relevant [55]. On the other hand, other studies reported similarities between the two investigated miRNA profiles, with minimal differences [21,57]. Altogether, these reports indicate that the source of liquid biopsy and the EV isolation method might have an effect on the individual assays and further downstream investigations, mostly in aspects regarding enrichment, proteic, and ribonucleic content and contaminants. These aspects will be discussed in the following section of this paper.

Up to this point, the composition and diagnostic potential of blood-derived exosomal miRNA signatures have been extensively described in the case of hepatocellular [58], prostate [59,60,61], ovarian [62,63], non-small-cell lung [53], colon [64,65], breast [66,67,68,69], gastrointestinal [70], and several other types of cancer [24]. Thus, it is beyond the goal of this paper to summarize all the identified exosomal miRNAs. We aim to offer an overview regarding novel methods applied in the investigation of exosomal miRNA from liquid biopsies, ranging from the use of alternative biofluids to the application and clinical utility of modern techniques in diagnosis, prognosis, and disease monitoring.

## 5. Characterization and Importance of Extracellular Vesicle (EV) miRNAs from Other Types of Liquid Biopsies

As mentioned previously, additional studies are required to validate the miRNA signature based on the respective biofluid of origin and cancer type. Biopsies from various biofluids might offer a different, more localized biomarker signature, as most investigated fluids have direct physiological interaction with the affected tissue. Therefore, we will focus on the EV miRNA signature identified in other biofluid specimens as alternative biomarker sources. Current studies focus on the extracellular vesicles mainly isolated from human blood, or, respectively, serum or plasma [71,72,73,74]. However, extracellular vesicles can be isolated from a broad variety of human body fluids such as urine [75,76], saliva [77,78], bronchoalveolar liquid [79], pleural lavage [80], and cerebrospinal fluid [81] and even more uncommon ones like tears [82], semen [83], menstrual blood [84], peritoneal lavage [85,86], bile [87], and pancreatic juice [88]. An overview of non-coding RNAs that were analyzed in extracellular vesicles isolated from different liquid biopsies of different kinds is presented in Table 1.

Not all approaches listed are related to cancer, though this overview might give an impression regarding the investigative possibilities of the respective biofluids in the context of novel cancer biomarker development. Some of the liquids mentioned are accessible without any invasive process such as urine, saliva, tears, or menstrual blood. Proximal fluids, however, cannot be obtained by a non-invasive or minimal-invasive procedure as a blood draw. Nonetheless, the majority of them are accrued during surgery such as peritoneal or pleural lavage [85,96]. Obtaining these kinds of liquid biopsies might be beneficial since body fluids derived from the proximity of the primary tumor bear the potential to better represent the molecular landscape of the tumor which would increase sensitivity and specificity of the diagnosis [87,96,103]. For example, EGFR genotyping of EVs of bronchoalveolar lavage fluid of NSCLC patients showed superior sensitivity and accuracy compared to plasma EV DNA genotyping [79]. These results suggest that this might also apply to ncRNA analysis of proximal fluids compared to EVs obtained from plasma or serum. Yet, apart from a few exceptions, a comparison of ncRNA from EVs of proximal fluids and serum/plasma is lacking. A comparison of human plasma and bronchoalveolar lavage (BAL) revealed a significantly lower concentration of EVs and miRNAs in the latter samples set. Since these results suggest that NSCLC tumor EVs are released into the blood rather than the bronchoalveolar liquid, BAL analysis does not show superior features in this context [95]. On the other hand, in a comparison of cholangiocarcinoma mRNA biomarkers derived from EVs of both urine and serum, more EV biomarkers were derived from serum, but urine EVs carried markers of diagnostic relevance that could not be found in serum EVs. Moreover, ncRNA was also found in urine-derived EVs that could accurately distinguish between patients with cholangiocarcinoma and primary sclerosing cholangitis. This implies that urine EVs could represent an important source of additional information for diagnosis [76].

The problem of a smaller number of biomarkers in EVs in comparison to blood is especially true regarding urine since glomerular filtration has an unexplored influence on EVs and their cargo [76]. Not only is the concentration of miRNAs of urine-derived EVs reported to be low, but so is the EV concentration itself [89]. Additionally, the exosome concentration is dependent on the urine volume which makes additional normalization steps necessary [105]. Still, other liquid biopsy samples harbor individual problems as well. A lower EV concentration in comparison with plasma was also reported for BAL samples [95]. Saliva is prone to bacterial contamination which has to be cleared before EV RNA analysis [77,78,106]. Pancreatic juice can be very viscous when patients suffer from intraductal papillary mucinous neoplasm due to high mucin concentration, which makes exosome isolation difficult [88]. High viscosity is also a problem reported for whole saliva [107]. These are just a few examples of problems that have to be overcome. Nonetheless, analyzing additional biofluids apart from blood might be beneficial as the following results suggest.

A comparison of diagnostic miRNA ratios in urine and serum in pancreatic cancer showed that the ratio was greater in EVs from urine, which leads to the hypothesis that urine might be a more suitable biomarker source than serum in this particular case [89]. Despite the problems mentioned before, most of the fluids analyzed so far can be processed similarly as blood. Very often, EVs are isolated by ultracentrifugation (UC) or similar to blood samples and only slight adaptations to the experimental protocols are necessary. Hence, comparing the sensitivity and specificity of classical liquid biopsy approaches with the use of proximal body fluid is still a gap yet to close. Up to now, the number of publications exploring the potential diagnostic value of EVs of liquid biopsies different from blood is rather limited, especially regarding ncRNA as biomarkers. A number of them represent proof of principle studies, investigating the general usability of different liquid biopsies to isolate EVs [77,83,104].

However, some of the studies could already demonstrate that analyzing miRNAs or long non-coding RNAs (lncRNAs) of EVs of other biofluids can discriminate between patients and healthy subjects [76,101,105], patients with different malignancies [76,88], or could show the importance of the additional diagnostic information EV analysis offers in comparison with standard diagnostic tools [87]. Therefore, analyzing EVs and especially ncRNAs from all of these kinds of liquid biopsies still bears great potential to be investigated in-depth and might represent interesting sources of information for cancer diagnosis and prognosis.

## 6. Technical Limitations of Current Methods

### 6.1. Isolation Methods—Standard and Advances

One of the main challenges in the clinical implementation of EVs and their cargo as biomarkers is the lack of standardization and consistency regarding their isolation methods. Current EV isolation methods can be technically challenging and laborious, with standard methods such as UC being generally regarded as the gold standard in the aspect of yield and purity. Therefore, a multitude of different methods used for the isolation of EVs from different body fluids has been developed to address these issues. Their strongpoints and limitations will be described.

### 6.2. Size and Polymer-Based Isolation Methods

The quality of exosomal miRNA profiles is heavily dependent on the methods used for the enrichment of EVs from the specific biological sample. Currently, the state-of-the-art method to isolate EVs from any kind of liquid biopsy is ultracentrifugation, or differential centrifugation [108]. However, this technique is time-consuming, labor-intense, and requires big sample volumes [108,109,110]. A method already employed on a more regular basis is EV precipitation [72,89,93]. Several comparison studies of these two methods exist. The comparison of the efficiency of EV isolation from human serum by UC and three commercial kits that are based on precipitation demonstrated that the isolation efficiency of UC is significantly lower compared to the precipitation kits, but this discrepancy was neither mirrored in the total exosomal RNA concentration nor its quality [108]. A similar study on plasma was performed which reports a significantly higher EV concentration in the samples processed by precipitation [111]. These results are further supported by another study also in favor of EV isolation by precipitation because a better reproducibility could be observed in samples isolated by this method [112]. Other authors claim that the decision for either method might be dependent on the sample number and availability of an ultracentrifuge [113]. Several other studies have reported differences regarding their yield and applicability for subsequent analysis of proteins, DNA, or miRNAs from several biological fluids [114,115,116]. Additionally, co-precipitations of protein aggregates, or albumin, is a common contaminant observed in the isolated exosomal fractions [114]. Polymer-based precipitation can provide some improvement to current precipitation methods. This method uses a copolymer to increase EV enrichment efficiency compared to UC and results in a fraction with less plasma protein contamination compared to commercial precipitation kits [110].

Due to the specific size of EVs of 30–150 nm, size-dependent isolation methods such as Size Exclusion Chromatography (SEC) are applicable. A porous polymer loaded column separates particles of different sizes in a solution. Particles bigger than the pore size, such as EVs, travel faster whereas smaller particles such as proteins are retained longer in the column and elute later. The pore size is adaptable by choosing the respective polymers. Mostly, different Sepharose matrices are used [117]. Comparison of EV isolation by SEC and UC from human plasma revealed, that the selection of the column matrix is crucial and has a great impact on the isolated EV fractions, regarding the amount of co-eluted albumin as well as EV size. The overall efficiency of the EV isolation by SEC was comparable to UC based isolation but was superior regarding albumin contamination [118]. Another study compared the two methods concerning their performance for EV isolation from human plasma. SEC outperformed UC regarding the isolation efficiency, however, samples showed significant contamination with albumin and the samples had the lowest abundance of other proteins that might be relevant for further analysis, as analyzed by mass spectrometry. A combination of one circle of UC and subsequent SEC could improve the results, but also resulted in increased albumin contamination compared to UC alone [119]. The comparatively high blood protein contamination is a common problem associated with SEC EV isolation since it has also been reported by another study analyzing rat blood plasma. Yet, also a high inter-experiment reproducibility for SEC is demonstrated [120]. Thus, due to its higher isolation efficiency and high reproducibility, SEC might represent an interesting alternative to UC, dependent on the downstream analysis. Additionally, in comparison to precipitation methods, the blood protein contamination is low, as demonstrated in a comparative study of SEC and precipitation agents for EV isolation from blood plasma, possibly making it a better alternative to UC than precipitation [121].

### 6.3. Microfluidic Chips

**Figure 1:**
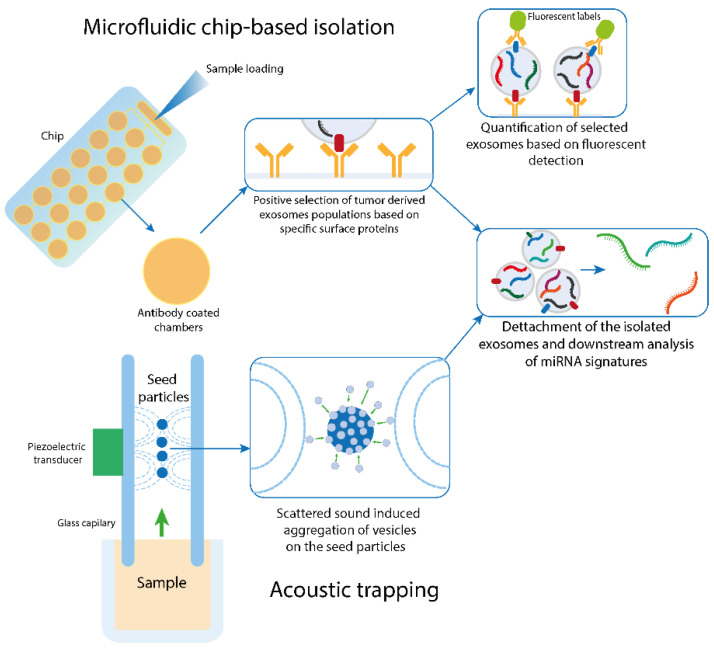
Overview of the principles of microfluidic and acoustic trapping EV isolation methods. Novel isolation methods can either use selective markers for isolations specific markers, such as the antibody-coated chips or rely on the size and physical properties of the analytes in the samples, such as the aggregation of the exosomes around seed particles following the entrapment by the scattered sound waves. Immunocapturing the exosomes can be informative regarding the specific populations of exosomes based on the utilized marker and a sandwich-type model employing additional fluorescent-labeled antibodies can be used for quantification. While the further applications, efficiency, and selective potential of these methods differ, the analysis of the miRNA signatures of the captured exosomes in both cases is done after their release from utilized isolation platform.

The advent of microfluidic technologies in recent years has also included potential novel methods for microvesicle isolation with increased reliability. Indirectly, these new methods would also facilitate the downstream analysis of their cargo, including the miRNA signature. Quite popular due to a broad spectrum of potential adaptations are Lab-on-the-Chip or Microfluidic techniques that utilize physical features of the EVs such as size or specific marker expression [122]. Chips that make use of the latter typically employ a support structure, to which capturing molecules are attached. This can be antibodies against tumor-derived markers as EpCAM [123], or exosome-specific surface proteins like CD9, CD63, and CD81 [124]. Another alternative target is Annexin V which binds phosphatidylserine, a component of the EV lipid layer [125]. Antibodies immobilized on the solid surfaces or magnetic beads capture the vesicles in the sample which can be furtherly removed and analyzed for their miRNA content [126] (Figure 1). Due to the free choice of capturing a molecule, these methods offer a broad application opportunity. The disadvantage is EVs not expressing the selected markers are captured by this isolation technique. To avoid this limitation, other approaches isolate exosomes based on their particular size. Several studies are applying different techniques. Assays that are merely size based mainly consist of two filters of different sizes to discard cell debris in the first step and protein in the second step of filtration [127,128]. Another method traps EVs between micropillars with silicon nanowires [129]. However, the majority of chips combine size-based captures by three-dimensional structures to increase the likelihood of the EVs to bind to the capture antibodies that are employed in many of these chips [130,131,132]. Other methods like membrane-based filtration or electrophoresis-driven filtrations all have provided promising results, yet a further method validation is necessary until any of them can be implemented at a clinical level. An extensive description of different microfluidic-based exosome isolation methods and their clinical potential is presented in another review [133].

### 6.4. Acoustic Trapping

One of the most recently developed methods is the acoustic trap. This approach uses a piezoelectric transducer to generate a local standing wave in an adjacent glass capillary. Seeding beads are suspended in the glass capillary due to secondary acoustic forces and act as aggregation points for the EVs in the sample. The sample is aspirated in the capillary and the so-called acoustic trap aggregates the EVs along with the seeding beads, which can be furtherly removed and the EVs separated for further investigation (Figure 1). The feasibility of acoustic trapping to isolate EVs from plasma was initially shown in 2015 [134] and later compared to classical isolation by UC. The latter remains the superior method in terms of the total yield of EVs. This might be due to several impact factors such as sample density and viscosity that can hamper isolation efficiency by acoustic trapping. Still, the same miRNAs could be found in comparable concentrations of EVs in urine and serum samples in a comparison of both isolation methods. This suggests that the amount of EVs isolated by acoustic trapping might be sufficient for diagnostic purposes [135]. Another study revealed that the RNA yield of EVs from an equivalent of 1.7 mL urine is sufficient to generate a NEXTflex cDNA library for Illumina Next Generation Sequencing (NGS) of miRNAs. Hence, this method represents a fast, automated alternative to ultracentrifugation [109,135]. The method was further improved by combining an acoustic trap and a microfluidic chip. This makes the isolation from EVs from multi-component body fluids like blood or saliva feasible since the flow rate in addition to the acoustic frequency can be adjusted to isolate specific subpopulations of the sample [107,136]. Naturally, every method has its drawbacks such as a high antibody demand for the immunoaffinity isolation, the dependency of sample density of the acoustic trap, or the lower specificity of size-based methods. Despite that, they overcome problems associated with EV isolation by UC since they require smaller sample volumes, are feasible for high-throughput screening, and are faster, making them interesting alternatives that need to be validated to make them accessible for clinical use. An overview of EV isolation methods including their advantages and disadvantages mentioned before are summarized in Table 2.

### 6.5. miRNA Detection from EV/Exosomes

Conventional methods in evaluating miRNA signatures of vesicles include sequencing [50,138] and RT-qPCR validation. Yet, the main limitation regarding current methods in miRNA isolation from exosomes extracted from most biofluids is a low concentration, possibly not sufficient for downstream applications. The use of commercial kits is reported in some cases to offer varying results [50], yet further validation is required for this aspect.

**Figure 2:**
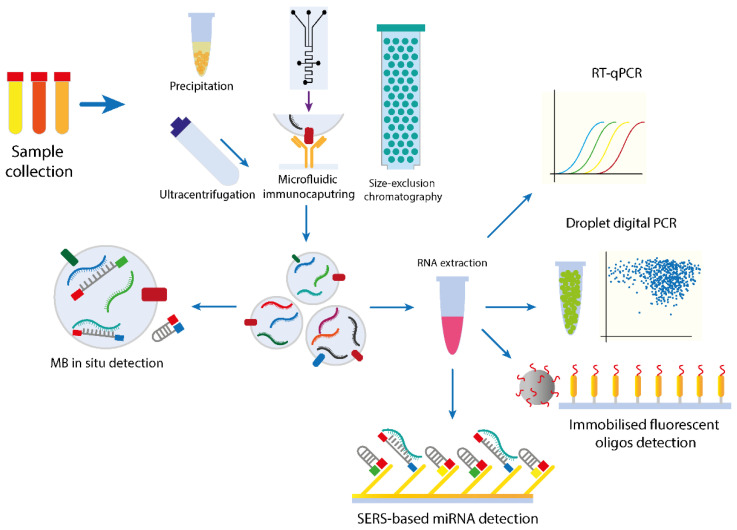
Overview of the described methods for characterization and quantification of exosomal miRNA. The workflow of exosomal miRNA detection and characterization varies based on the methods used for isolation, the efficiency, and yields of which have been previously described. Following this, most methods rely on the prerequisite RNA isolation from the exosomes to be analyzed using either amplification-based assays (RT-PCR or ddPCR) or oligo-based fluorescent or Surface-Enhanced Raman Scattering (SERS)-based techniques that can offer a more direct quantification. In situ detection methods can overcome the biological limitation imposed by the exosome lipid membrane and can detect the target miRNA inside the exosomes.

Droplet digital PCR is a recent technological improvement of the classical RT-qPCR that addresses the problem of sensitivity in the detection of lower transcript copy numbers. The basic principle of ddPCR consists of the partition of the reaction volume into thousands of oil droplets that encapsulates all the reagents but only one single template copy. The amplification is done in parallel, and the fluorescent signal originating from each droplet is individually measured. Additionally, ddPCR provides information regarding the endpoint of the reaction, thus indicating the absolute presence or absence of the transcript of interest even at lower initial copy numbers [139] (Figure 2).

Previous comparisons of the two PCR methods in evaluating circulating miRNA reported a higher precision and improved reproducibility of ddPCR for serum miRNA [140]. In the case of exosomal miRNA detection, ddPCR has been used in a study investigating the performance in comparison with classical RT-qPCR of urine-derived exosomal miRNAs. The group reported significant differences regarding minimal detectable template concentrations. Specifically, EV derived miR-29 was detected at less than 50 copies/μL using ddPCR, while RT-PCR required at least 6473 copies/μL [141]. Another study focused on the technical differences between several commercial serum exosome isolation kits and their impact of subsequent ddPCR analysis, for determining underlying variations in miRNA signatures resulted from each kit. The levels of the investigated miRNAs, miR-16 and miR-451 varied slightly based on the utilized kit, but the overall reliability of the extraction and detection methods indicated consistent results [108]. Other species of exosomal RNA have been investigated using ddPCR. Total exosomal RNA derived from plasma collected from castration-resistant prostate cancer (CRPC) patients was analyzed to detect androgen receptor splice variant 7 (AR-V7) mRNA, a factor associated with hormonal therapy resistance. The group confirmed that AR-V7 mRNA can be sensibly detected using ddPCR from plasma exosomes, providing a useful biomarker in predicting therapeutic resistance in CRPC [142].

Recently, PCR free techniques emerged as possible alternatives to classical miRNA determination methods. These techniques generally make use of different localized electrochemical or optical physical processes as a base for the development of novel biosensors. A new method utilizes a redox system to electrochemically detect exosomal miRNAs that are adsorbed on oligo-functionalized magnetic beads. Specifically, isolated exosomal miRNAs were captured using biotinylated probes on streptavidin-coated magnetic beads. The purified miRNAs were then adsorbed on a bare gold surface with screen-printed electrodes. The miRNA quantification was done by measuring the electron transfer between the localized redox system formed by the gold surface and [Fe(CN)_6_]^4−/3−^. The proof of concept consisted of the analysis of miR-21 levels isolated from colorectal carcinoma (CRC) patients and cell lines, respectively. The method indicated high reproducibility and sensibility based on PCR validation and serial dilution evaluation of detection limit, which indicated minimal concentration of 1.0 pM as a limit of detection (LOD) [143].

A thermophoresis-based method that utilizes special constructs called NanoFlares for the detection of exosomal miRNAs has been recently described [144]. NanoFlares consist of gold nanoparticles that are functionalized with fluorescent DNA oligos that could capture the target miRNA in situ, meaning that there is no requirement of prerequisite RNA extraction and/or amplification. Hybridization of the target miRNA with the oligos on the Nanoflares produces a fluorescent signal, which can be thermophoretically amplified by localized laser-induced heating. The method was tested on serum-isolated exosomes originating from ER-breast cancer patients. The NanoFlares were functionalized with oligos specific for a set of four BC-specific miRNAs, specifically miR-375, miR-221, miR-210, and miR-10b with which exosomal miRNAs from the sample could be detected with a minimum of 83% specificity. The group indicated that the minimal miRNA detection amount was 0.36 fM and a minimal serum sample volume of 0.5 μL [144].

The need for multiplex detection methods has emerged as more studies report that disease-specific miRNA signatures are composed of multiple miRNAs. Thus, the simultaneous identification of multiple cancer-related miRNA biomarkers can result in more specific and time-efficient screening methods. A multiplex PCR-free method of exosomal miRNA detection in situ used fluorescent molecular beacons (MBs) [145]. Molecular beacons (MBs) consist of hairpin-shaped fluorescent oligos that are quenched in their native unbound state due to the proximity of the quencher to the fluorophore. The hybridization of the MB with a target sequence distances the quencher from the fluorophore, emitting the specific signal in an on/off switch-like manner (Figure 2). The group confirmed that using three non-overlapping spectra fluorescent oligo beacons for the multiplex detection of exosomal miR-375, miR-21, and miR-27a, the signal could still be detected in the presence of serum without additional treatments [145]. Yet, it is not mentioned whether circulating miRNA could interact with the MB when in complete serum. The method was subsequently extended to also include the immunodetection of exosomal proteins that could facilitate exosome detection. Anti-CD63 magnetic beads were used to positively select pancreatic cell line-derived exosomes that were furtherly analyzed using previously described MB for determining miR-21 localization. Additionally, the group proposed the use of additional exosomal surface biomarkers, such as EpCAM, EGFR, survivin, and IGF-1R as pancreatic cancer-associated markers. The fluorescent values from the detection of the surface markers were normalized by the fluorescent intensity of CD63, the initial selection marker for the exosomes [146]. Although the method provided promising results, there are many aspects regarding normalization, the applicability in human biofluid samples, and the accessibility of the detection methods, which require further development. The same group utilized an MB-based method for the detection of prostate cancer-associated miRNA from both cell lines and human urine. The method allowed the in situ single or dual detection of miRNA in the isolated exosomes with high specificity, indicating that urine is a suitable biofluid for miRNA signature analysis using this method and emphasized that miRNA can be detected in the exosome without previous extraction [147]. A good example of the use of MBs coupled with silver nanorod RAMAN substrate was utilized in constructing an array for the multiplex detection of several lung-cancer associated miRNAs, namely miRNA-21, miRNA-486, and miRNA-375. The designed MB for each miRNA were immobilized on the Surface-Enhanced Raman Scattering (SERS) substrate, which allowed the hybridization of the miRNA present in the sample [148]. The measurements resulting from the analysis of both miRNA solutions with known concertation and human serum confirmed the accuracy of the method, providing good insights about the clinical implementations of similar methods.

A recently developed electrochemical exosomal miRNA assay utilized DNA-hairpin probes on a gold electrode to detect target minimal amounts of miRNA. The method relied on the signal amplification, resulting from a hybridization chain reaction (HCR). While exosome isolation and RNA extraction were performed separately, the group investigated the presence of miR-122 in their sample. The outline of the method consists of the binding of the target miRNA to the hairpin DNA probes immobilized on the gold electrodes. Following the duplex formation, HCR is triggered by hybridizing additional DNA hairpins that facilitate the capture of RuHex, an electrochemical signal reporter. Since it is an electroactive substance, binding to the DNA backbone results in an amplification of the electrochemical signal. According to the authors, this method is highly sensitive, cheap, and results are reproducible. However, their assay was not used yet to analyze patient samples [149].

## 7. Applications of Surface-Enhanced Raman Scattering (SERS) Based Techniques in EV miRNA Detection

Optical sensor-based technologies have recently tried to address the problems regarding the lack of sensitivity of the current biomarker detection methods associated with the low analyte concentrations in the investigated biological samples [150].

Raman scattering is a process based on the vibrational signature of individual chemical bonds following photonic excitation [151]. This allows the identification of specific molecules based on vibrational fingerprint, offering information about the components in a mixture, such as a biological sample. Yet, Raman scattering is normally a rarely occurring process, as only one in a million photons scatter inelastically. Therefore, methods of amplifying the scattering signal have concluded that by adsorbing the investigated molecule on a metal surface, the signal is increased drastically and allows for the detection of lower concentrated analytes. The technical adaptation of this method resulted in the Surface-Enhanced Raman Scattering (SERS), a Raman based spectroscopy that employs the use of optically characterized Au or Ag nanostructures in the form of 2D nanostructures or colloidal nanoparticles to amplify the scattering signal (Figure 2). Metallic particles provide enhancing properties to the detectable signal due to a combination of electromagnetic and chemical effects [152]. The extension of SERS for biomedical use required additional methodological adaptations that allowed the detection of DNA, proteins, miRNAs, and exosomes from a variety of biofluids [153,154]. The detection of biological molecules is usually done using either an unlabeled or labeled approach. The label-free SERS detection is based on the direct adsorption of the molecule of interest on the used metallic nanostructure or particle followed by the detection of the spectra based on the vibrational signature [154]. One of the first SERS-based miRNA detection systems developed more than a decade ago utilized a label-free sequence-dependent method to distinguish different miRNAs with high accuracy. The group utilized an oblique angle vapor deposition (OAD) method to create specially aligned silver nanorod arrays the would be used as SERS substrates for miRNA detection. Following this, the group was able to identify and differentiate between five synthetized unrelated miRNAs and all the members of the let-7 mRNA family based on the SERS spectral signature determined by the vibrational signature corresponding to their nucleotide composition [155]. However, while the method could differentiate between miRNAs at one nucleotide level, no further development of this method for clinical use has been employed at this moment.

The development of high precision exosomal miRNA detection is required to overcome several limitations imposed by the biological nature of the analyte. Particularly, as the investigated miRNAs are encapsulated, MB, or other oligo-based techniques such as the ones described previously are not suitable for the detection. Thus, the collaborative approach between efficient exosomal isolation with more sensible miRNA detection methods might provide specific insight regarding the potential use of exosomal miRNAs as reliable biomarkers.

### 7.1. SERS-Based Exosome Detection and Quantification

**Figure 3:**
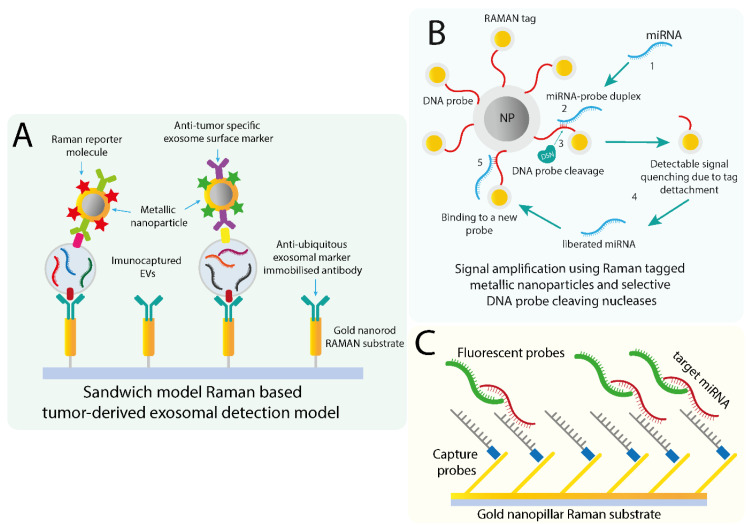
Overview of several SERS-based exosomes (**A**) and miRNA detection and quantification methods utilizing both metallic nanoparticle (**B**) and immobilized (**C**) Raman substrates. The developed SERS-based methods are focused on the low amounts of the analyte, either whole exosomes or exons. The sandwich-model (**A**) is focused on the SERS detection of specific exosome populations disregarding their miRNA cargo by the use of antibody-coupled SERS reporter molecules. For exomiRs, the described SERS substrates address the low isolation yield limitation by utilizing signal-amplifying approaches in conjunction with detectable Raman tags (**B**) or a combination of dual-targeting capturing substrates with fluorescent probes (**C**).

More recent methods employ labels in the form of reporter molecules with specific spectroscopic Raman signals that coat metallic nanostructures. Additionally, the reporter molecules are used in conjunction with specific targeting molecules, such as antibodies in the case of vesicle identification or oligos for miRNA that create specific recognition assays. As a general overview, current exosomal detection methods are dependent on the selection of the exosomes based on their surface protein markers. First, an exosomal selection marker is generally used, such as CD63, to immunocapture the vesicles on a metallic structure [154]. Then, specific SERS tags in the form of metallic nanoparticles are conjugated with antibodies specific for a cancer-specific protein marker (Figure 3A). This is described as a sandwich-model detection method, which can be functionalized for multiplexing by adding additional SERS tags that could detect multiple tumor markers. As a relevant example of a SERS tag-based technique, a group utilized magnetic beads coated with anti-CD63 antibodies as a ubiquitous exosomal marker. In conjunction with anti-HER2 coated Gold-Silver nanorods, they acted both as a tumor-derived exosome indicator and RAMAN reporter molecule. This resulted in a sandwich-type model that allowed to specifically discern tumor-associated exosomes from exosome secreted from normal cell lines [156]. In a later study, the same group utilized reporter molecules conjugated with additional tumor markers antibodies to propose a multiplex exosomal surface protein detection method that takes into consideration the heterogeneity of EV populations [157]. A similar sandwich model was applied in the development of bi-functionalized SERS immunoassay named PEARL (polydopamine-encapsulated antibody-reporter-Ag(shell)–Au(core) multilayer), which utilized migration inhibitory factor (MIF) as the main marker associated with pancreatic cell-derived exosomes [158]. Based on the identification of MIF, the immunosensor differentiated healthy from PC patients. Additionally, MIF PEARL SERS tags efficiently distinguished between metastatic and non-metastatic patients. It is important to mention that the analysis was done using 2 µL of clinical serum that required no additional pre-treatment or exosome enrichment.

### 7.2. Detection of EV/Exosomal miRNA Using SERS Based Methods

In comparison with the early miRNA identification methods, more methods have shifted onto a SERS tagging approach that could improve the specificity and multiplexing capability. One study investigated the miRNA signature isolated from exosomes using an adapted dual SERS biosensor. The sensor utilized Fe_3_O_4_@Ag as a SERS substrate and designed Fe_3_O_4_@Ag-DNA-Au@Ag@DTNB SERS tags with DNA oligos complementary to the target miRNA sequences. The method relied on the hybridization of the target miRNA with the DNA oligo on the SERS tagged nanoparticle, followed by the selective cleavage of the DNA from the miRNA/DNA duplex by a duplex-specific nuclease (DSN). The cleavage induces the separation of the SERS tag from the substrate and the associated quenching can be detected (Figure 3B). This process is repeated thousands of times, as the miRNA is not cleaved by the DSN, allowing the amplification of the signal for identifying that specific miRNA to a detection limit of 1 aM [159]. The group furtherly investigated the expression level of miRNA-10b from plasma-derived exosomes and supernatant plasma obtained from pancreatic ductal adenocarcinoma and chronic prostatitis patients and validated that the method could accurately differentiate between the two groups and the healthy control group [159]. A variant of this method was previously employed to characterize exosomal miRNA extracted from non-small-cell lung adenocarcinoma patients’ plasma. This variant utilized a SERS signal reporter called ARANPs, gold nanoparticles conjugated with the R6G fluorophore partially coated in an AgAu alloy. Silicone separating substrate microbeads were used for attaching the 5′ end of the DNA target probe, while the ARNAPs were bound to the 3′ end of the probe. As in the previously described method, signal was based on the DSN cleavage activity and target-recycling amplification. The method was validated by measuring plasma exosomal-derived miR-21 from NSCLC patients and healthy control. The group reported significantly elevated miR-21 levels in the NSCLC patient group, which was consistent with the RT-qPCR results. Similarly, another group developed a locked nucleic acid (LNA) based gold nanopillar SERS substrate that also detected exosomal miRNA with similar detection range and specificity. The method consisted of constructing a gold nanopillar SERS substrate that was decorated with fluorophore-labeled oligos specific for the target miRNA (Figure 3C). In this case, the group analyzed a set of three miRNA, miR-21, miR-222, and miR-200c extracted from breast cancer cell lines. The group indicated that based on the expression levels of these miRNA, the method could differentiate between BC subtypes based on the detection of these exosomal miRNAs, thus emphasizing the clinical value of both exosomal miRNAs and this method in breast cancer diagnosis and subtyping [160].

## 8. Conclusions

One of the few ways of making liquid biopsies more efficient is to maximize the potential information provided about the pathology by a combination of specific biomarkers and sensible methods. Liquid biopsy, through its minimal-invasiveness, can provide real-time information about diagnostics, disease staging, and therapeutic efficiency. Yet, the need for standardization in the area of liquid biopsy biomarkers identifiable is still limited by a series of obstacles.

MiRNAs play an important role in cancer progression and their specific signatures in the case of many types of cancer have given rise to a plethora of studies investigating their biomarker potential. In our case, EV derived miRNAs, or exomiRs, represent an interesting source of information since they are loaded into the EVs by tumor cells and are protected for degradation and can provide sensible cancer-specific signatures [46,47,48,55]. Nonetheless, the main limitation in the use of extracellular vesicles (e.g., exosomes) is the lack of consistency regarding the applied methods used for their isolation and characterization.

Furthermore, this review provides an overview of different liquid biopsy specimens. Up to date, the majority of publications analyze EV cargo from serum and plasma. However, EVs are abundant and can be isolated from any kind of body fluid. Researchers were able to use ncRNA from EVs of unconventional specimens to discriminate between cancer patients and healthy individuals with high accuracy. Thus, validation of these liquid biopsies is required as well as comparisons with serum or plasma EVs to verify them as additional sources of information for clinical use.

While the problem of specimen has been addressed, we underlined the utility of technical adaptations of classic methods, such as ddPCR or fluorescent-based detection methods as they either provide quicker analysis [127] or feasibility for high-throughput screening [107] and require a smaller screening volume. If these techniques are further improved and the advantages combined, they might make implementing EV isolation in clinical settings possible. Furthermore, the clinical implementation of SERS based methods could provide powerful tools in the detection of specific biomarkers. Their applicability in the detection of exomiRs and both exosomes and miRNA separately have provided promising results in minimal sample volumes. Taking all the recent advances together, it shows that there is a lot of work done regarding the improvement of the steps necessary to exploit the clinically valuable information carried by EVs. Hence, a translation of a mere laboratory technique into an application in a clinical setting might be possible within the next few years.

## Figures and Tables

**Table 1:** Overview of specimen-derived extracellular vesicles (EVs) for cargo analysis.

Specimen	Non-Coding RNA	Malignancy	Isolation Method	Comparison with Plasma/Serum EV Analysis	Reference
Urine	lncRNA; miRNA,	Cholangiocarcinoma; Pancreatic Ductal Adenocarcinoma; Breast cancer; Bladder cancer Endometrial Cancer	Ultra-centrifugation, ExoQuick, Nano-membrane concentrator	+ non-invasive + low cost − fewer biomarkers found − low concentration of EVs in urine	[75,76,89,90,91]
Saliva	miRNA	Oral Squamous Cell Carcinoma	Ultra-centrifugation	+ no coagulation + non-invasive − Viral/bacterial contamination	[77,78,92]
Broncho-alveolar liquid	miRNA; lncRNA	Lung adenocarcinoma, NSCLC	Ultra-centrifugation ExoQuick	+ higher accuracy due to site specification − lower concentration of exosomes	[79,93,94,95]
Pleural lavage	miRNA	Lung Cancer	miRCURY Exosome isolation Kit Ultra-centrifugation	+ additional diagnostic and prognostic information + potentially higher sensitivity and specificity	[80,96,97]
Cerebro- spinal liquid	miRNA	Glioma	Ultra-centrifugation	+ higher sensitivity − More invasive	[98,99]
Tears		Breast cancer	Nanocavity platform	+ easy, non-invasive collection + no pre-treatment necessary	[82]
Semen	miRNA	Prostate cancer	Ultra-centrifugation ddPCR		[83,100,101]
Menstrual blood	miRNA (therapeutic)	Pulmonary Fibrosis	Ultra-centrifugation		[84]
Peritoneal lavage/ascitic fluid	miRNA	Endometrial cancer; Colorectal Cancer; Gastric cancer	Ultra-centrifugation	+ better representation of the molecular landscape of tumor − Difficult to obtain	[85,86,102,103]
Bile	miRNA	Cholangio-carcinoma	Ultra-centrifugation		[87]
Pancreatic Juice	miRNA	Pancreatic Ductal Adenocarcinoma	CD63 labeled magnetic beads, Ultra-centrifugation	+ potentially diagnostically more accurate due to direct contact to tumor	[88,104]

**Table 2:** Overview of the advantages and disadvantages of described EV isolation methods.

Method	Principle	Advantage	Disadvantage	Reference
Ultra-centrifugation	Stepwise removal of other components by centrifugation	High purity	Labor-intense Time-consuming Inconsistent results High sample volume required	[108,111,112,113]
Precipitation	Making use of the low water solubility of EVs	Better reproducibility than UC Fast	Co-precipitation of proteins Lower isolation efficiency	[108,111,112,113]
Size Exclusion Chromatography	Particles in a solution flow through a polymer filled column in a different time, dependent on their size	Higher isolation efficiency than UC Better reproducibility than UC	High blood protein contamination	[117,118,119,120,121]
Size Based Chip	Excluding bigger compartments like CTCs and smaller like debris by dual filtration	Independent of marker expression Improved purity Low cost Low sample volume required Fast	Clogging of the system occurs Device fabrication necessary	[127,128,129]
Marker Based Chip	Tether EVs by specific markers expressed on the surface	Wide range of sample volume Better reproducibility Low cost Applicable for High-Throughput application Fast Potential to individualize	Loss of EVs that do not carry the marker Device fabrication necessary Off-Chip steps required Antibody demand might be high	[123,125,131,137]
Acoustic Trap	Trap EVs by capture beads in a standing wave induced by a piezoelectric transducer	Better reproducibility Improved purity Increased isolation efficiency Fast Adjustable particle size Low sample volume required	A lower yield of EVs than with UC Complex technical set-up	[109,134,135,136]

## References

[1] Chanteloup G., Cordonnier M., Isambert N., Bertaut A., Hervieu A., Hennequin A., Luu M., Zanetta S., Coudert B., Bengrine L. (2020). Monitoring HSP70 exosomes in cancer patients’ follow up: A clinical prospective pilot study. J. Extracell. Vesicles.

[2] Ko J.M.-Y., Vardhanabhuti V., Ng W.-T., Lam K.-O., Ngan R.K.-C., Kwong D.L.-W., Lee V.H., Lui Y.-H., Yau C.-C., Kwan C.-K. (2020). Clinical utility of serial analysis of circulating tumour cells for detection of minimal residual disease of metastatic nasopharyngeal carcinoma. Br. J. Cancer.

[3] Alix-Panabières C. (2020). The future of liquid biopsy. Nature.

[4] Lianidou E., Pantel K. (2019). Liquid biopsies. Genes Chromosomes Cancer.

[5] Kowalik A., Kowalewska M., Góźdź S. (2017). Current approaches for avoiding the limitations of circulating tumor cells detection methods-implications for diagnosis and treatment of patients with solid tumors. Transl. Res. J. Lab. Clin. Med..

[6] Lim S.B., Di Lee W., Vasudevan J., Lim W.-T., Lim C.T. (2019). Liquid biopsy: One cell at a time. NPJ Precis. Oncol..

[7] Bronkhorst A.J., Ungerer V., Holdenrieder S. (2019). The emerging role of cell-free DNA as a molecular marker for cancer management. Biomol. Detect. Quantif..

[8] Corcoran R.B., Chabner B.A. (2018). Application of cell-free DNA analysis to cancer treatment. N. Engl. J. Med..

[9] Johansson G., Andersson D., Filges S., Li J., Muth A., Godfrey T.E., Ståhlberg A. (2019). Considerations and quality controls when analyzing cell-free tumor DNA. Biomol. Detect. Quantif..

[10] Fici P., Casadio V., Salvi S. (2019). Cell-free DNA in the liquid biopsy context: Role and differences between ctDNA and CTC marker in cancer management. Cell-Free DNA as Diagnostic Markers: Methods and Protocols.

[11] Khier S., Lohan L. (2018). Kinetics of circulating cell-free DNA for biomedical applications: Critical appraisal of the literature. Future Sci. OA.

[12] Fabbri M., Calin G.A. (2010). Epigenetics and miRNAs in Human Cancer. Adv. Genet..

[13] Pardini B., Sabo A.A., Birolo G., Calin G.A. (2019). Noncoding RNAs in Extracellular Fluids as Cancer Biomarkers: The New Frontier of Liquid Biopsies. Cancers.

[14] Chaubey A., Karanti S., Rai D., Oh T., Adhvaryu S.G., Aguiar R.C.T. (2009). microRNAs and deletion of the derivative chromosome 9 in chronic myeloid leukemia. Leukemia.

[15] Wilting S.M., Snijders P.J.F., Verlaat W., Jaspers A., van de Wiel M.A., van Wieringen W.N., Meijer G.A., Kenter G.G., Yi Y., le Sage C. (2013). Altered microRNA expression associated with chromosomal changes contributes to cervical carcinogenesis. Oncogene.

[16] Huppi K., Volfovsky N., Runfola T., Jones T.L., Mackiewicz M., Martin S.E., Mushinski J.F., Stephens R., Caplen N.J. (2008). The identification of microRNAs in a genomically unstable region of human chromosome 8q24. Mol. Cancer Res..

[17] Ding C., Chen S.-N., Macleod R.A.F., Drexler H.G., Nagel S., Wu D.-P., Sun A.-N., Dai H.-P. (2018). miR-130a is aberrantly overexpressed in adult acute myeloid leukemia with t(8;21) and its suppression induces AML cell death. Ups. J. Med. Sci..

[18] Kurahashi R., Kadomatsu T., Baba M., Hara C., Itoh H., Miyata K., Endo M., Morinaga J., Terada K., Araki K. (2019). microRNA-204-5p: A novel candidate urinary biomarker of Xp11.2 translocation renal cell carcinoma. Cancer Sci..

[19] Hirata Y., Murai N., Yanaihara N., Saito M., Saito M., Urashima M., Murakami Y., Matsufuji S., Okamoto A. (2014). microRNA-21 is a candidate driver gene for 17q23-25 amplification in ovarian clear cell carcinoma. BMC Cancer.

[20] Lee J.-H., List A., Sallman D.A. (2019). Molecular pathogenesis of myelodysplastic syndromes with deletion 5q. Eur. J. Haematol..

[21] Rabinowits G., Gerçel-Taylor C., Day J.M., Taylor D.D., Kloecker G.H. (2009). Exosomal microRNA: A Diagnostic Marker for Lung Cancer. Clin. Lung Cancer.

[22] Bullock M.D., Silva A.M., Kanlikilicer-Unaldi P., Filant J., Rashed M.H., Sood A.K., Lopez-Berestein G., Calin G.A. (2015). Exosomal non-coding RNAs: Diagnostic, prognostic and therapeutic applications in cancer. Non-Coding RNA.

[23] Mitchell P.S., Parkin R.K., Kroh E.M., Fritz B.R., Wyman S.K., Pogosova-Agadjanyan E.L., Peterson A., Noteboom J., O’Briant K.C., Allen A. (2008). Circulating microRNAs as stable blood-based markers for cancer detection. Proc. Natl. Acad. Sci. USA.

[24] Yang B., Xiong W.-Y., Hou H.-J., Xu Q., Cai X.-L., Zeng T.-X., Ha X.-Q. (2019). Exosomal miRNAs as biomarkers of cancer: A meta-analysis. Clin. Lab..

[25] Cornell L., Wander S.A., Visal T., Wagle N., Shapiro G.I. (2019). microRNA-mediated suppression of the TGF-β pathway confers transmissible and reversible CDK4/6 inhibitor resistance. Cell Rep..

[26] Cai X., Qu L., Yang J., Xu J., Sun L., Wei X., Qu X., Bai T., Guo Z., Zhu Y. (2020). Exosome-transmitted microRNA-133b inhibited bladder cancer proliferation by upregulating dual-specificity protein phosphatase 1. Cancer Med..

[27] Malla R.R., Pandrangi S., Kumari S., Gavara M.M., Badana A.K. (2018). Exosomal tetraspanins as regulators of cancer progression and metastasis and novel diagnostic markers. Asia Pac. J. Clin. Oncol..

[28] Bobrie A., Colombo M., Krumeich S., Raposo G., Théry C. (2012). Diverse subpopulations of vesicles secreted by different intracellular mechanisms are present in exosome preparations obtained by differential ultracentrifugation. J. Extracell. Vesicles.

[29] Valadi H., Ekström K., Bossios A., Sjöstrand M., Lee J.J., Lötvall J.O. (2007). Exosome-mediated transfer of mRNAs and microRNAs is a novel mechanism of genetic exchange between cells. Nat. Cell Biol..

[30] Wang L., Li Y., Guan X., Zhao J., Shen L., Liu J. (2018). Exosomal double-stranded DNA as a biomarker for the diagnosis and preoperative assessment of pheochromocytoma and paraganglioma. Mol. Cancer.

[31] Yokoi A., Villar-Prados A., Oliphint P.A., Zhang J., Song X., Hoff P.D., Morey R., Liu J., Roszik J., Clise-Dwyer K. (2019). Mechanisms of nuclear content loading to exosomes. Sci. Adv..

[32] Takahashi A., Okada R., Nagao K., Kawamata Y., Hanyu A., Yoshimoto S., Takasugi M., Watanabe S., Kanemaki M.T., Obuse C. (2017). Exosomes maintain cellular homeostasis by excreting harmful DNA from cells. Nat. Commun..

[33] Sansone P., Savini C., Kurelac I., Chang Q., Amato L.B., Strillacci A., Stepanova A., Iommarini L., Mastroleo C., Daly L. (2017). Packaging and transfer of mitochondrial DNA via exosomes regulate escape from dormancy in hormonal therapy-resistant breast cancer. Proc. Natl. Acad. Sci. USA.

[34] Zhang H., Deng T., Liu R., Bai M., Zhou L., Wang X., Li S., Wang X., Yang H., Li J. (2017). Exosome-delivered EGFR regulates liver microenvironment to promote gastric cancer liver metastasis. Nat. Commun..

[35] Jurj A., Pop L., Petrushev B., Pasca S., Dima D., Frinc I., Deak D., Desmirean M., Trifa A., Fetica B. (2018). Exosome-carried microRNA-based signature as a cellular trigger for the evolution of chronic lymphocytic leukemia into Richter syndrome. Crit. Rev. Clin. Lab. Sci..

[36] Zhan Y., Du L., Wang L., Jiang X., Zhang S., Li J., Yan K., Duan W., Zhao Y., Wang L. (2018). Expression signatures of exosomal long non-coding RNAs in urine serve as novel non-invasive biomarkers for diagnosis and recurrence prediction of bladder cancer. Mol. Cancer.

[37] Wang J., Pu J., Zhang Y., Yao T., Luo Z., Li W., Xu G., Liu J., Wei W., Deng Y. (2020). Exosome-transmitted long non-coding RNA SENP3-EIF4A1 suppresses the progression of hepatocellular carcinoma. Aging.

[38] Mathieu M., Martin-Jaular L., Lavieu G., Théry C. (2019). Specificities of secretion and uptake of exosomes and other extracellular vesicles for cell-to-cell communication. Nat. Cell Biol..

[39] Greening D.W., Ji H., Chen M., Robinson B.W.S., Dick I.M., Creaney J., Simpson R.J. (2016). Secreted primary human malignant mesothelioma exosome signature reflects oncogenic cargo. Sci. Rep..

[40] Kobayashi M., Rice G.E., Tapia J., Mitchell M.D., Salomon C. Exosomes are Fingerprints of Originating Cells: Potential Biomarkers for Ovarian Cancer. https://www.dovepress.com/exosomes-are-fingerprints-of-originating-cells-potential-biomarkers-fo-peer-reviewed-fulltext-article-RRBC.

[41] Lobb R.J., Hastie M.L., Norris E.L., van Amerongen R., Gorman J.J., Möller A. (2017). Oncogenic transformation of lung cells results in distinct exosome protein profile similar to the cell of origin. Proteomics.

[42] Sanz-Rubio D., Martin-Burriel I., Gil A., Cubero P., Forner M., Khalyfa A., Marin J.M. (2018). Stability of circulating exosomal miRNAs in healthy subjects. Sci. Rep..

[43] Science Advances Mechanisms of Nuclear Content Loading to Exosomes. https://advances.sciencemag.org/content/5/11/eaax8849.

[44] Djebali S., Davis C.A., Merkel A., Dobin A., Lassmann T., Mortazavi A., Tanzer A., Lagarde J., Lin W., Schlesinger F. (2012). Landscape of transcription in human cells. Nature.

[45] Anfossi S., Fu X., Nagvekar R., Calin G.A. (2018). MicroRNAs, Regulatory Messengers Inside and Outside Cancer Cells. Neurodegener. Dis..

[46] Gibbings D.J., Ciaudo C., Erhardt M., Voinnet O. (2009). Multivesicular bodies associate with components of miRNA effector complexes and modulate miRNA activity. Nat. Cell Biol..

[47] Kosaka N., Iguchi H., Yoshioka Y., Takeshita F., Matsuki Y., Ochiya T. (2010). Secretory mechanisms and intercellular transfer of microRNAs in living cells. J. Biol. Chem..

[48] Kosaka N., Iguchi H., Hagiwara K., Yoshioka Y., Takeshita F., Ochiya T. (2013). Neutral sphingomyelinase 2 (nSMase2)-dependent exosomal transfer of angiogenic microRNAs regulate cancer cell metastasis. J. Biol. Chem..

[49] Nik Mohamed Kamal N.N.S.B., Shahidan W.N.S. (2020). Non-exosomal and exosomal circulatory microRNAs: Which are more valid as biomarkers?. Front. Pharmacol..

[50] Cheng L., Sun X., Scicluna B.J., Coleman B.M., Hill A.F. (2014). Characterization and deep sequencing analysis of exosomal and non-exosomal miRNA in human urine. Kidney Int..

[51] Ge Q., Zhou Y., Lu J., Bai Y., Xie X., Lu Z. (2014). miRNA in plasma exosome is stable under different storage conditions. Mol. Basel Switz..

[52] Endzeliņš E., Berger A., Melne V., Bajo-Santos C., Soboļevska K., Ābols A., Rodriguez M., Šantare D., Rudņickiha A., Lietuvietis V. (2017). Detection of circulating miRNAs: Comparative analysis of extracellular vesicle-incorporated miRNAs and cell-free miRNAs in whole plasma of prostate cancer patients. BMC Cancer.

[53] Liu Q., Yu Z., Yuan S., Xie W., Li C., Hu Z., Xiang Y., Wu N., Wu L., Bai L. (2017). Circulating exosomal microRNAs as prognostic biomarkers for non-small-cell lung cancer. Oncotarget.

[54] Wu Q., Yu L., Lin X., Zheng Q., Zhang S., Chen D., Pan X., Huang Y. (2020). Combination of serum miRNAs with serum exosomal miRNAs in early diagnosis for non-small-cell lung cancer. Cancer Manag. Res..

[55] Cheng L., Sharples R.A., Scicluna B.J., Hill A.F. (2014). Exosomes provide a protective and enriched source of miRNA for biomarker profiling compared to intracellular and cell-free blood. J. Extracell. Vesicles.

[56] Tian F., Shen Y., Chen Z., Li R., Ge Q. (2017). No significant difference between plasma miRNAs and plasma-derived exosomal miRNAs from healthy people. BioMed Res. Int..

[57] Taylor D.D., Gercel-Taylor C. (2008). microRNA signatures of tumor-derived exosomes as diagnostic biomarkers of ovarian cancer. Gynecol. Oncol..

[58] Wang Y., Zhang C., Zhang P., Guo G., Jiang T., Zhao X., Jiang J., Huang X., Tong H., Tian Y. (2018). Serum exosomal microRNAs combined with alpha-fetoprotein as diagnostic markers of hepatocellular carcinoma. Cancer Med..

[59] Valentino A., Reclusa P., Sirera R., Giallombardo M., Camps C., Pauwels P., Crispi S., Rolfo C. (2017). Exosomal microRNAs in liquid biopsies: Future biomarkers for prostate cancer. Clin. Transl. Oncol. Off. Publ. Fed. Span. Oncol. Soc. Natl. Cancer Inst. Mex..

[60] Bhagirath D., Yang T.L., Bucay N., Sekhon K., Majid S., Shahryari V., Dahiya R., Tanaka Y., Saini S. (2018). microRNA-1246 is an exosomal biomarker for aggressive prostate cancer. Cancer Res..

[61] Malla B., Zaugg K., Vassella E., Aebersold D.M., Dal Pra A. (2017). Exosomes and exosomal microRNAs in prostate cancer radiation therapy. Int. J. Radiat. Oncol. Biol. Phys..

[62] Pan C., Stevic I., Müller V., Ni Q., Oliveira-Ferrer L., Pantel K., Schwarzenbach H. (2018). Exosomal microRNAs as tumor markers in epithelial ovarian cancer. Mol. Oncol..

[63] Giannopoulou L., Zavridou M., Kasimir-Bauer S., Lianidou E.S. (2019). Liquid biopsy in ovarian cancer: The potential of circulating miRNAs and exosomes. Transl. Res..

[64] Ogata-Kawata H., Izumiya M., Kurioka D., Honma Y., Yamada Y., Furuta K., Gunji T., Ohta H., Okamoto H., Sonoda H. (2014). Circulating exosomal microRNAs as biomarkers of colon cancer. PLoS ONE.

[65] Hosseini M., Khatamianfar S., Hassanian S.M., Nedaeinia R., Shafiee M., Maftouh M., Ghayour-Mobarhan M., ShahidSales S., Avan A. (2017). Exosome-encapsulated microRNAs as potential circulating biomarkers in colon cancer. Curr. Pharm. Des..

[66] Hannafon B.N., Trigoso Y.D., Calloway C.L., Zhao Y.D., Lum D.H., Welm A.L., Zhao Z.J., Blick K.E., Dooley W.C., Ding W.Q. (2016). Plasma exosome microRNAs are indicative of breast cancer. Breast Cancer Res. BCR.

[67] He Y., Deng F., Yang S., Wang D., Chen X., Zhong S., Zhao J., Tang J. (2018). Exosomal microRNA: A novel biomarker for breast cancer. Biomark. Med..

[68] Rodríguez-Martínez A., de Miguel-Pérez D., Ortega F.G., García-Puche J.L., Robles-Fernández I., Exposito J., Martorell-Marugan J., Carmona-Sáez P., Garrido-Navas M.D.C., Rolfo C. (2019). Exosomal miRNA profile as complementary tool in the diagnostic and prediction of treatment response in localized breast cancer under neoadjuvant chemotherapy. Breast Cancer Res. BCR.

[69] Joyce D.P., Kerin M.J., Dwyer R.M. (2016). Exosome-encapsulated microRNAs as circulating biomarkers for breast cancer. Int. J. Cancer.

[70] Nedaeinia R., Manian M., Jazayeri M.H., Ranjbar M., Salehi R., Sharifi M., Mohaghegh F., Goli M., Jahednia S.H., Avan A. (2017). Circulating exosomes and exosomal microRNAs as biomarkers in gastrointestinal cancer. Cancer Gene Ther..

[71] De Miguel Pérez D., Rodriguez Martínez A., Ortigosa Palomo A., Delgado Ureña M., Garcia Puche J.L., Robles Remacho A., Exposito Hernandez J., Lorente Acosta J.A., Ortega Sánchez F.G., Serrano M.J. (2020). Extracellular vesicle-miRNAs as liquid biopsy biomarkers for disease identification and prognosis in metastatic colorectal cancer patients. Sci. Rep..

[72] Ozawa P.M.M., Vieira E., Lemos D.S., Souza I.L.M., Zanata S.M., Pankievicz V.C., Tuleski T.R., Souza E.M., Wowk P.F., de Urban C.A. (2020). Identification of miRNAs enriched in extracellular vesicles derived from serum samples of breast cancer patients. Biomolecules.

[73] Kanlikilicer P., Bayraktar R., Denizli M., Rashed M.H., Ivan C., Aslan B., Mitra R., Karagoz K., Bayraktar E., Zhang X. (2018). Exosomal miRNA confers chemo resistance via targeting Cav1/p-gp/M2-type macrophage axis in ovarian cancer. EBioMedicine.

[74] Qu L., Ding J., Chen C., Wu Z.-J., Liu B., Gao Y., Chen W., Liu F., Sun W., Li X.-F. (2016). Exosome-transmitted lncARSR promotes sunitinib resistance in renal cancer by acting as a competing endogenous RNA. Cancer Cell.

[75] Gonzales P.A., Zhou H., Pisitkun T., Wang N.S., Star R.A., Knepper M.A., Yuen P.S.T. (2010). Isolation and purification of exosomes in urine. Methods Mol. Biol. Clifton NJ.

[76] Lapitz A., Arbelaiz A., O’Rourke C.J., Lavin J.L., La Casta A., Ibarra C., Jimeno J.P., Santos-Laso A., Izquierdo-Sanchez L., Krawczyk M. (2020). Patients with cholangiocarcinoma present specific RNA profiles in serum and urine extracellular vesicles mirroring the tumor expression: Novel liquid biopsy biomarkers for disease diagnosis. Cells.

[77] Michael A., Bajracharya S., Yuen P., Zhou H., Star R., Illei G., Alevizos I. (2010). Exosomes from human saliva as a source of microRNA biomarkers. Oral Dis..

[78] Gai C., Camussi F., Broccoletti R., Gambino A., Cabras M., Molinaro L., Carossa S., Camussi G., Arduino P.G. (2018). Salivary extracellular vesicle-associated miRNAs as potential biomarkers in oral squamous cell carcinoma. BMC Cancer.

[79] Hur J.Y., Lee J.S., Kim I.A., Kim H.J., Kim W.S., Lee K.Y. (2019). Extracellular vesicle-based EGFR genotyping in bronchoalveolar lavage fluid from treatment-naive non-small cell lung cancer patients. Transl. Lung Cancer Res..

[80] Watabe S., Kikuchi Y., Morita S., Komura D., Numakura S., Kumagai-Togashi A., Watanabe M., Matsutani N., Kawamura M., Yasuda M. (2020). Clinicopathological significance of microRNA-21 in extracellular vesicles of pleural lavage fluid of lung adenocarcinoma and its functions inducing the mesothelial to mesenchymal transition. Cancer Med..

[81] Bounajem M.T., Karsy M., Jensen R.L. (2020). Liquid biopsies for the diagnosis and surveillance of primary pediatric central nervous system tumors: A review for practicing neurosurgeons. Neurosurg. Focus.

[82] Takeuchi T., Mori K., Sunayama H., Takano E., Kitayama Y., Shimizu T., Hirose Y., Inubushi S., Sasaki R., Tanino H. (2020). Antibody-conjugated signaling nanocavities fabricated by dynamic molding for detecting cancers using small extracellular vesicle markers from tears. J. Am. Chem. Soc..

[83] Vojtech L., Woo S., Hughes S., Levy C., Ballweber L., Sauteraud R.P., Strobl J., Westerberg K., Gottardo R., Tewari M. (2014). Exosomes in human semen carry a distinctive repertoire of small non-coding RNAs with potential regulatory functions. Nucleic Acids Res..

[84] Sun L., Zhu M., Feng W., Lin Y., Yin J., Jin J., Wang Y. (2019). Exosomal miRNA Let-7 from menstrual blood-derived endometrial stem cells alleviates pulmonary fibrosis through regulating mitochondrial DNA damage. Oxid. Med. Cell. Longev..

[85] Roman-Canal B., Moiola C.P., Gatius S., Bonnin S., Ruiz-Miró M., González E., González-Tallada X., Llordella I., Hernández I., Porcel J.M. (2019). EV-associated miRNAs from peritoneal lavage are a source of biomarkers in endometrial cancer. Cancers.

[86] Ohzawa H., Kumagai Y., Yamaguchi H., Miyato H., Sakuma Y., Horie H., Hosoya Y., Kawarai Lefor A., Sata N., Kitayama J. (2020). Exosomal microRNA in peritoneal fluid as a biomarker of peritoneal metastases from gastric cancer. Ann. Gastroenterol. Surg..

[87] Li L., Masica D., Ishida M., Tomuleasa C., Umegaki S., Kalloo A.N., Georgiades C., Singh V.K., Khashab M., Amateau S. (2014). Human bile contains microRNA-laden extracellular vesicles that can be used for cholangiocarcinoma diagnosis. Hepatology.

[88] Nakamura S., Sadakari Y., Ohtsuka T., Okayama T., Nakashima Y., Gotoh Y., Saeki K., Mori Y., Nakata K., Miyasaka Y. (2019). Pancreatic juice exosomal microRNAs as biomarkers for detection of pancreatic ductal adenocarcinoma. Ann. Surg. Oncol..

[89] Yoshizawa N., Sugimoto K., Tameda M., Inagaki Y., Ikejiri M., Inoue H., Usui M., Ito M., Takei Y. (2020). miR-3940-5p/miR-8069 ratio in urine exosomes is a novel diagnostic biomarker for pancreatic ductal adenocarcinoma. Oncol. Lett..

[90] Ando W., Kikuchi K., Uematsu T., Yokomori H., Takaki T., Sogabe M., Kohgo Y., Otori K., Ishikawa S., Okazaki I. (2019). Novel breast cancer screening: Combined expression of miR-21 and MMP-1 in urinary exosomes detects 95% of breast cancer without metastasis. Sci. Rep..

[91] Srivastava A., Moxley K., Ruskin R., Dhanasekaran D.N., Zhao Y.D., Ramesh R. (2018). A Non-invasive liquid biopsy screening of urine-derived exosomes for miRNAs as biomarkers in endometrial cancer patients. AAPS J..

[92] Elashoff D., Zhou H., Reiss J., Wang J., Henson B., Hu S., Arellano M., Sinha U., Le A., Messadi D. (2012). Pre-validation of salivary biomarkers for oral cancer detection. Cancer Epidemiol. Biomark. Prev. Publ. Am. Assoc. Cancer Res. Cosponsored Am. Soc. Prev. Oncol..

[93] Kim J.E., Eom J.S., Kim W., Jo E.J., Mok J., Lee K., Kim K.U., Park H., Lee M.K., Kim M. (2018). Diagnostic value of microRNAs derived from exosomes in bronchoalveolar lavage fluid of early-stage lung adenocarcinoma: A pilot study. Thorac. Cancer.

[94] Wu F., Yin Z., Yang L., Fan J., Xu J., Jin Y., Yu J., Zhang D., Yang G. (2019). Smoking induced extracellular vesicles release and their distinct properties in non-small cell lung cancer. J. Cancer.

[95] Rodríguez M., Silva J., López-Alfonso A., López-Muñiz M.B., Peña C., Domínguez G., García J.M., López-Gónzalez A., Méndez M., Provencio M. (2014). Different exosome cargo from plasma/bronchoalveolar lavage in non-small-cell lung cancer. Genes Chromosomes Cancer.

[96] Roman-Canal B., Moiola C.P., Gatius S., Bonnin S., Ruiz-Miró M., González E., Ojanguren A., Recuero J.L., Gil-Moreno A., Falcón-Pérez J.M. (2019). EV-associated miRNAs from pleural lavage as potential diagnostic biomarkers in lung cancer. Sci. Rep..

[97] Hydbring P., De Petris L., Zhang Y., Brandén E., Koyi H., Novak M., Kanter L., Hååg P., Hurley J., Tadigotla V. (2018). Exosomal RNA-profiling of pleural effusions identifies adenocarcinoma patients through elevated miR-200 and LCN2 expression. Lung Cancer.

[98] Shi R., Wang P.-Y., Li X.-Y., Chen J.-X., Li Y., Zhang X.-Z., Zhang C.-G., Jiang T., Li W.-B., Ding W. (2015). Exosomal levels of miRNA-21 from cerebrospinal fluids associated with poor prognosis and tumor recurrence of glioma patients. Oncotarget.

[99] Akers J.C., Ramakrishnan V., Kim R., Phillips S., Kaimal V., Mao Y., Hua W., Yang I., Fu C.-C., Nolan J. (2015). miRNA contents of cerebrospinal fluid extracellular vesicles in glioblastoma patients. J. Neurooncol..

[100] Vickram A.S., Samad H.A., Latheef S.K., Chakraborty S., Dhama K., Sridharan T.B., Sundaram T., Gulothungan G. (2020). Human prostasomes an extracellular vesicle—Biomarkers for male infertility and prostrate cancer: The journey from identification to current knowledge. Int. J. Biol. Macromol..

[101] Barceló M., Castells M., Bassas L., Vigués F., Larriba S. (2019). Semen miRNAs contained in exosomes as non-invasive biomarkers for prostate cancer diagnosis. Sci. Rep..

[102] Yamamoto C.M., Oakes M.L., Murakami T., Muto M.G., Berkowitz R.S., Ng S.-W. (2018). Comparison of benign peritoneal fluid- and ovarian cancer ascites-derived extracellular vesicle RNA biomarkers. J. Ovarian Res..

[103] Roman-Canal B., Tarragona J., Moiola C.P., Gatius S., Bonnin S., Ruiz-Miró M., Sierra J.E., Rufas M., González E., Porcel J.M. (2019). EV-associated miRNAs from peritoneal lavage as potential diagnostic biomarkers in colorectal cancer. J. Transl. Med..

[104] Osteikoetxea X., Benke M., Rodriguez M., Pálóczi K., Sódar B.W., Szvicsek Z., Szabó-Taylor K., Vukman K.V., Kittel Á., Wiener Z. (2018). Detection and proteomic characterization of extracellular vesicles in human pancreatic juice. Biochem. Biophys. Res. Commun..

[105] Matsuzaki K., Fujita K., Jingushi K., Kawashima A., Ujike T., Nagahara A., Ueda Y., Tanigawa G., Yoshioka I., Ueda K. (2017). miR-21-5p in urinary extracellular vesicles is a novel biomarker of urothelial carcinoma. Oncotarget.

[106] Nonaka T., Wong D.T.W. (2017). Saliva-exosomics in cancer: Molecular characterization of cancer-derived exosomes in saliva. Enzymes.

[107] Wang Z., Li F., Rufo J., Chen C., Yang S., Li L., Zhang J., Cheng J., Kim Y., Wu M. (2020). Acoustofluidic salivary exosome isolation: A liquid biopsy compatible approach for human papillomavirus–associated oropharyngeal cancer detection. J. Mol. Diagn..

[108] Helwa I., Cai J., Drewry M.D., Zimmerman A., Dinkins M.B., Khaled M.L., Seremwe M., Dismuke W.M., Bieberich E., Stamer W.D. (2017). A comparative study of serum exosome isolation using differential ultracentrifugation and three commercial reagents. PLoS ONE.

[109] Ku A., Ravi N., Yang M., Evander M., Laurell T., Lilja H., Ceder Y. (2019). A urinary extracellular vesicle microRNA biomarker discovery pipeline; from automated extracellular vesicle enrichment by acoustic trapping to microRNA sequencing. PLoS ONE.

[110] Zhong Z., Rosenow M., Xiao N., Spetzler D. (2018). Profiling plasma extracellular vesicle by pluronic block-copolymer based enrichment method unveils features associated with breast cancer aggression, metastasis and invasion. J. Extracell. Vesicles.

[111] Serrano-Pertierra E., Oliveira-Rodríguez M., Rivas M., Oliva P., Villafani J., Navarro A., Blanco-López M.C., Cernuda-Morollón E. (2019). Characterization of plasma-derived extracellular vesicles isolated by different methods: A comparison study. Bioengineering.

[112] Caradec J., Kharmate G., Hosseini-Beheshti E., Adomat H., Gleave M., Guns E. (2014). Reproducibility and efficiency of serum-derived exosome extraction methods. Clin. Biochem..

[113] Alvarez M.L., Khosroheidari M., Kanchi Ravi R., DiStefano J.K. (2012). Comparison of protein, microRNA, and mRNA yields using different methods of urinary exosome isolation for the discovery of kidney disease biomarkers. Kidney Int..

[114] Ding M., Wang C., Lu X., Zhang C., Zhou Z., Chen X., Zhang C.-Y., Zen K., Zhang C. (2018). Comparison of commercial exosome isolation kits for circulating exosomal microRNA profiling. Anal. Bioanal. Chem..

[115] Chevillet J.R., Kang Q., Ruf I.K., Briggs H.A., Vojtech L.N., Hughes S.M., Cheng H.H., Arroyo J.D., Meredith E.K., Gallichotte E.N. (2014). Quantitative and stoichiometric analysis of the microRNA content of exosomes. Proc. Natl. Acad. Sci. USA.

[116] Royo F., Zuñiga-Garcia P., Sanchez-Mosquera P., Egia A., Perez A., Loizaga A., Arceo R., Lacasa I., Rabade A., Arrieta E. (2016). Different EV enrichment methods suitable for clinical settings yield different subpopulations of urinary extracellular vesicles from human samples. J. Extracell. Vesicles.

[117] Monguió-Tortajada M., Gálvez-Montón C., Bayes-Genis A., Roura S., Borràs F.E. (2019). Extracellular vesicle isolation methods: Rising impact of size-exclusion chromatography. Cell. Mol. Life Sci..

[118] Baranyai T., Herczeg K., Onódi Z., Voszka I., Módos K., Marton N., Nagy G., Mäger I., Wood M.J., El Andaloussi S. (2015). Isolation of exosomes from blood plasma: Qualitative and quantitative comparison of ultracentrifugation and size exclusion chromatography methods. PLoS ONE.

[119] An M., Wu J., Zhu J., Lubman D.M. (2018). Comparison of an optimized ultracentrifugation method versus size-exclusion chromatography for isolation of exosomes from human serum. J. Proteome Res..

[120] Takov K., Yellon D.M., Davidson S.M. (2018). Comparison of small extracellular vesicles isolated from plasma by ultracentrifugation or size-exclusion chromatography: Yield, purity and functional potential. J. Extracell. Vesicles.

[121] Gámez-Valero A., Monguió-Tortajada M., Carreras-Planella L., Franquesa M., Beyer K., Borràs F.E. (2016). Size-exclusion chromatography-based isolation minimally alters extracellular vesicles’ characteristics compared to precipitating agents. Sci. Rep..

[122] Liga A., Vliegenthart A.D.B., Oosthuyzen W., Dear J.W., Kersaudy-Kerhoas M. (2015). Exosome isolation: A microfluidic road-map. Lab Chip.

[123] Dong J., Zhang R.Y., Sun N., Smalley M., Wu Z., Zhou A., Chou S.-J., Jan Y.J., Yang P., Bao L. (2019). Bio-inspired nanovilli chips for enhanced capture of tumor-derived extracellular vesicles: Toward non-invasive detection of gene alterations in non-small cell lung cancer. ACS Appl. Mater. Interfaces.

[124] Kamyabi N., Abbasgholizadeh R., Maitra A., Ardekani A., Biswal S.L., Grande-Allen K.J. (2020). Isolation and mutational assessment of pancreatic cancer extracellular vesicles using a microfluidic platform. Biomed. Microdevices.

[125] Kang Y.-T., Purcell E., Palacios-Rolston C., Lo T.-W., Ramnath N., Jolly S., Nagrath S. (2019). Isolation and profiling of circulating tumor-associated exosomes using extracellular vesicular lipid–protein binding affinity based microfluidic device. Small.

[126] Kanwar S.S., Dunlay C.J., Simeone D.M., Nagrath S. (2014). Microfluidic device (ExoChip) for on-chip isolation, quantification and characterization of circulating exosomes. Lab Chip.

[127] Sunkara V., Kim C.-J., Park J., Woo H.-K., Kim D., Ha H.K., Kim M.-H., Son Y., Kim J.-R., Cho Y.-K. (2019). Fully automated, label-free isolation of extracellular vesicles from whole blood for cancer diagnosis and monitoring. Theranostics.

[128] Liu F., Vermesh O., Mani V., Ge T.J., Madsen S.J., Sabour A., Hsu E.-C., Gowrishankar G., Kanada M., Jokerst J.V. (2017). The exosome total isolation chip. ACS Nano.

[129] Wang Z., Wu H., Fine D., Schmulen J., Hu Y., Godin B., Zhang J.X.J., Liu X. (2013). Ciliated micropillars for the microfluidic-based isolation of nanoscale lipid vesicles. Lab Chip.

[130] Zhang P., Zhou X., He M., Shang Y., Tetlow A.L., Godwin A.K., Zeng Y. (2019). Ultrasensitive detection of circulating exosomes with a 3D-nanopatterned microfluidic chip. Nat. Biomed. Eng..

[131] Zhou S., Hu T., Zhang F., Tang D., Li D., Cao J., Wei W., Wu Y., Liu S. (2020). Integrated microfluidic device for accurate extracellular vesicle quantification and protein markers analysis directly from human whole blood. Anal. Chem..

[132] Reátegui E., van der Vos K.E., Lai C.P., Zeinali M., Atai N.A., Aldikacti B., Floyd F.P., Khankhel A.H., Thapar V., Hochberg F.H. (2018). Engineered nanointerfaces for microfluidic isolation and molecular profiling of tumor-specific extracellular vesicles. Nat. Commun..

[133] Contreras-Naranjo J.C., Wu H.-J., Ugaz V.M. (2017). Microfluidics for exosome isolation and analysis: Enabling liquid biopsy for personalized medicine. Lab Chip.

[134] Evander M., Gidlöf O., Olde B., Erlinge D., Laurell T. (2015). Non-contact acoustic capture of microparticles from small plasma volumes. Lab Chip.

[135] Ku A., Lim H.C., Evander M., Lilja H., Laurell T., Scheding S., Ceder Y. (2018). Acoustic enrichment of extracellular vesicles from biological fluids. Anal. Chem..

[136] Wu M., Ouyang Y., Wang Z., Zhang R., Huang P.-H., Chen C., Li H., Li P., Quinn D., Dao M. (2017). Isolation of exosomes from whole blood by integrating acoustics and microfluidics. Proc. Natl. Acad. Sci. USA.

[137] Zhao Z., Yang Y., Zeng Y., He M. (2016). A microfluidic ExoSearch chip for multiplexed exosome detection towards blood-based ovarian cancer diagnosis. Lab Chip.

[138] Cook G.W., Benton M.G., Akerley W., Mayhew G.F., Moehlenkamp C., Raterman D., Burgess D.L., Rowell W.J., Lambert C., Eng K. (2020). Structural variation and its potential impact on genome instability: Novel discoveries in the EGFR landscape by long-read sequencing. PLoS ONE.

[139] Pinheiro L.B., Coleman V.A., Hindson C.M., Herrmann J., Hindson B.J., Bhat S., Emslie K.R. (2012). Evaluation of a droplet digital polymerase chain reaction format for DNA copy number quantification. Anal. Chem..

[140] Hindson C.M., Chevillet J.R., Briggs H.A., Gallichotte E.N., Ruf I.K., Hindson B.J., Vessella R.L., Tewari M. (2013). Absolute quantification by droplet digital PCR versus analog real-time PCR. Nat. Methods.

[141] Wang C., Ding Q., Plant P., Basheer M., Yang C., Tawedrous E., Krizova A., Boulos C., Farag M., Cheng Y. (2019). Droplet digital PCR improves urinary exosomal miRNA detection compared to real-time PCR. Clin. Biochem..

[142] Del Re M., Biasco E., Crucitta S., Derosa L., Rofi E., Orlandini C., Miccoli M., Galli L., Falcone A., Jenster G.W. (2017). The detection of androgen receptor splice variant 7 in plasma-derived exosomal RNA strongly predicts resistance to hormonal therapy in metastatic prostate cancer patients. Eur. Urol..

[143] Boriachek K., Umer M., Islam M.N., Gopalan V., Lam A.K., Nguyen N.-T., Shiddiky M.J.A. (2018). An amplification-free electrochemical detection of exosomal miRNA-21 in serum samples. Analyst.

[144] Zhao J., Liu C., Li Y., Ma Y., Deng J., Li L., Sun J. (2020). Thermophoretic detection of exosomal microRNAs by nanoflares. J. Am. Chem. Soc..

[145] Lee J.H., Kim J.A., Jeong S., Rhee W.J. (2016). Simultaneous and multiplexed detection of exosome microRNAs using molecular beacons. Biosens. Bioelectron..

[146] Cho S., Yang H.C., Rhee W.J. (2019). Simultaneous multiplexed detection of exosomal microRNAs and surface proteins for prostate cancer diagnosis. Biosens. Bioelectron..

[147] Lee J., Kwon M.H., Kim J.A., Rhee W.J. (2018). Detection of exosome miRNAs using molecular beacons for diagnosing prostate cancer. Artif. Cells Nanomedicine Biotechnol..

[148] Song C.Y., Yang Y.J., Yang B.Y., Sun Y.Z., Zhao Y.P., Wang L.H. (2016). An ultrasensitive SERS sensor for simultaneous detection of multiple cancer-related miRNAs. Nanoscale.

[149] Guo Q., Yu Y., Zhang H., Cai C., Shen Q. (2020). Electrochemical sensing of exosomal microRNA based on hybridization chain reaction signal amplification with reduced false-positive signals. Anal. Chem..

[150] Ray P., Steckl A.J. (2019). Label-free optical detection of multiple biomarkers in sweat, plasma, urine, and saliva. ACS Sens..

[151] Raman C.V. (1928). A new radiation. Indian J. Phys..

[152] Li W., Zhao X., Yi Z., Glushenkov A.M., Kong L. (2017). Plasmonic substrates for surface enhanced Raman scattering. Anal. Chim. Acta.

[153] Blanco-Formoso M., Alvarez-Puebla R.A. (2020). Cancer diagnosis through SERS and other related techniques. Int. J. Mol. Sci..

[154] Zhang Y., Mi X., Tan X., Xiang R. (2019). Recent progress on liquid biopsy analysis using surface-enhanced raman spectroscopy. Theranostics.

[155] Driskell J.D., Seto A.G., Jones L.P., Jokela S., Dluhy R.A., Zhao Y.-P., Tripp R.A. (2008). Rapid microRNA (miRNA) detection and classification via surface-enhanced Raman spectroscopy (SERS). Biosens. Bioelectron..

[156] Zong S., Wang L., Chen C., Lu J., Zhu D., Zhang Y., Wang Z., Cui Y. (2016). Facile detection of tumor-derived exosomes using magnetic nanobeads and SERS nanoprobes. Anal. Methods.

[157] Wang Z., Zong S., Wang Y., Li N., Li L., Lu J., Wang Z., Chen B., Cui Y. (2018). Screening and multiple detection of cancer exosomes using an SERS-based method. Nanoscale.

[158] Li T.-D., Zhang R., Chen H., Huang Z.-P., Ye X., Wang H., Deng A.-M., Kong J.-L. (2018). An ultrasensitive polydopamine bi-functionalized SERS immunoassay for exosome-based diagnosis and classification of pancreatic cancer. Chem. Sci..

[159] Pang Y., Wang C., Lu L., Wang C., Sun Z., Xiao R. (2019). Dual-SERS biosensor for one-step detection of microRNAs in exosome and residual plasma of blood samples for diagnosing pancreatic cancer. Biosens. Bioelectron..

[160] Lee J.U., Kim W.H., Lee H.S., Park K.H., Sim S.J. (2019). Quantitative and specific detection of exosomal miRNAs for accurate diagnosis of breast cancer using a surface-enhanced Raman scattering sensor based on plasmonic head-flocked gold nanopillars. Small.

